# Case Report: A novel germline donor splicing site mutation of *RB1* gene in a Chinese Tibetan pedigree with familial retinoblastoma

**DOI:** 10.3389/fonc.2025.1525035

**Published:** 2025-05-20

**Authors:** Guo-qian He, Ying-chun Zheng, Lin-jun Tan, Cheng-qi Shen, Ju Gao, Fu Xiong, Xia Guo

**Affiliations:** ^1^ Department of Pediatrics, West China Second University Hospital, Sichuan University, Chengdu, Sichuan, China; ^2^ Key Laboratory of Birth Defects and Related Diseases of Women and Children (Sichuan University), Ministry of Education, Chengdu, Sichuan, China; ^3^ Department of Medical Genetics, School of Basic Medical Sciences, Southern Medical University, Guangzhou, China; ^4^ Department of Pediatrics, Affiliated Hospital of Zunyi Medical University, Zunyi, China; ^5^ NHC Key Laboratory of Chronobiology, Sichuan University, Chengdu, China

**Keywords:** retinoblastoma, *RB1 gene*, donor splicing site mutation, germline, child

## Abstract

Retinoblastoma (RB) is the most common primary intraocular malignancy in children and mostly initiates with biallelic inactivation of the *RB1* gene. Hereditary retinoblastoma accounts for 40% of all cases, with only 6%–10% of patients having a positive family history. The proband, a Chinese Tibetan boy, was diagnosed with RB for leukocoria. The *RB1* gene mutations were screened due to disease recurrence. A novel germline donor splicing site mutation (c.861 + 2T>A) from his father was identified by Sanger sequencing and a novel somatic duplication mutation in exon 2 221-224 (p.W75Cfs*36) by next-generation sequencing (NGS). The proband’s younger brother manifested bilateral RB and also carried the same germline mutation. To further explore the possible pathogenicity of the novel germline *RB1* mutation (c.861 + 2T>A) in RB development, mutation analysis, bioinformatics analysis, and immunohistochemistry were performed. After *RB1* cDNA was amplified, the abnormal script was found to be smaller than the normal script. Compared with normal samples, Sanger sequencing revealed a deletion of 143 bp in the abnormal script. In comparison to healthy individuals, patients exhibited a reduction in the mRNA expression levels of the *RB1* gene. The three-dimensional structure predicted by iterative threading assembly refinement (I-TASSER) indicates significant changes in the spatial structure of abnormal proteins after mutation. No expression of RB1 was found in tumor tissue by immunohistochemistry evaluation. Therefore, the novel germline donor splicing site mutation (c.861 + 2T>A) has been confirmed to be a pathological mutation.

## 1 Introduction

Retinoblastoma (RB), the most prevalent primary intraocular cancer in children, has a global estimated annual incidence rate ranging from 1 in 15,000 to 1 in 20,000 live births, accounting for 2.5% to 4% of pediatric tumors (1, 2). According to the Global Retinoblastoma Study Group, the incidence of retinoblastoma varies by region, with Asia reporting the highest number of cases at 52%, followed by Africa (23.6%), Latin America (7.6%), North America (4.5%), and other regions. This distribution is influenced by population size and birth rates (3, 4). In Europe, a higher estimate of the incidence of RB was reported, with an incidence of 1 in 13,844 live births, or 14.1 and 4.6 cases per 1 million children aged under 5 and 15 years, respectively (5). Poor outcome correlates with delayed “lag time”, difficulty in accessing retinoblastoma-specific healthcare, and socioeconomic issues leading to poor compliance, including refusal of enucleation and abandonment of treatment (6, 7). In high-income countries, unilateral disease is typically diagnosed at approximately 2 years of age, whereas bilateral disease is diagnosed at a median age of 1 year. The age at diagnosis is approximately doubled in countries with lower national incomes (2). Mean overall and disease-free survival rates also vary considerably depending on socioeconomic status, ranging from less than 50% in low- and middle-income countries to over 90% in high-income countries (3, 8).

The two-hit hypothesis was formulated by Knudson that RB requires loss-of-function of tumor suppressor *RB1* gene owing to homozygous allelic mutations, mechanism of loss of heterozygosity (LOH), or gene silencing. Since retinoma, a benign retinal lesion, has also undergone loss of both RB1^−/−^ alleles, biallelic inactivation of RB1 is crucial for initiating most RB cases, yet it alone is insufficient for malignancy (9). Further genetic or epigenetic changes are likely needed for malignant transformation. Motivated by the observation that genomic gains and LOH are often present in addition to biallelic *RB1* gene inactivation, a multi-step model for the development of RB has been proposed (7, 10, 11). Studies have evidenced copy number variations (CNVs) in retinoblastoma, including gains of 1q, 2p, 6p, and 13q and loss of 13q and 16q, which delineate areas of the genome where oncogenes or tumor suppressors may lie. Notably, *KIFI4*, *MDM4*, *MYCN*, *E2F3*, *CDHI1*, *RBL2*, and *CREEBP* may all be candidate genes. Moreover, recurrent single-nucleotide variants (SNVs) in the *BCOR* gene and aberrant methylation of certain promoters such as those of *MGMT*, *RASSF1A*, *CASP8*, and *MLH1* genes also participate in the mutational landscape. The *RB1* mutation types, the methylation status of its promoter, and the accompanying somatic anomalies in the mutational landscape are thought to define together the aggressiveness of the disease (2, 12).

Approximately 40% to 50% of individuals diagnosed with RB harbor a germline *RB1* mutation, with a significantly higher rate of 97% in patients with bilateral RB and 15% in those with unilateral RB (12, 13). Familial retinoblastoma accounts for a smaller percentage of all cases, with estimates ranging from 5% to 10% according to the Global Retinoblastoma Study Group (3). Due to the older age and cT4 advanced tumor as independent factors for worse survival, early screening of *RB1* gene mutation, especially in at-risk infants with a positive family history as soon as possible after birth, is the internationally accepted convention for RB (13, 15). Here, we report a Chinese Tibetan pedigree with RB carrying a novel germline *RB1* intron splicing site mutation. The proband had unilateral retinoblastoma with c.861 + 2T>A mutation, which had never been reported to date in RB. The proband’s father was blind for unknown reasons, and the proband’s younger brother had bilateral RB. Both of them carried the same germline *RB1* gene mutation. Further validation tests were carried out to confirm the mutation’s pathogenicity. These results may help clinicians deepen their understanding of familial RB.

## 2 Materials and methods

### 2.1 Patients

The proband, a boy aged 2 years 11 months, was diagnosed with RB. The proband was referred to the Department of Pediatric Hematology/Oncology at West China Second University Hospital of Sichuan University for chemotherapy. The Ethics Committee of West China Second University Hospital has granted authorization for this study. Furthermore, we commend the patient’s family for providing written informed consent, enabling us to proceed with the necessary medical procedures and research.

### 2.2 *RB1* gene mutation site screening

Genomic DNA was extracted from the peripheral blood of the proband and his family members by a standard phenol/chloroform extraction method. This process ensures the high quality and purity of the DNA samples for further analysis. Following the identification of potential RB1 gene mutation sites via Sanger sequencing, which covered exons 1–27, all family members were subjected to locus-specific amplification using polymerase chain reaction (PCR), followed by confirmation through Sanger sequencing (**Supplementary Table 1**). The primer sequences were as follows: forward primer: 5′-AGCAGAGTAGAAGAGGGATGGC and reverse primer: 5′-ACTTTTCAGTGATTCCAGAGTGAGG. Enucleation surgery was performed after tumor recurrence. In collaboration with Kindstar Globalgene Technology (Beijing, China), advanced genomic analysis was conducted on the tumor tissue of the proband, including next-generation sequencing (NGS) and whole-genome microarray analysis (CMA).

### 2.3 Bioinformatics

The pathogenic potential of c.861 + 2T>A, located in the intron shear region, was assessed using the MutationTaster software (http://www.mutationtaster.org/). The I-TASSER service was used to predict the three-dimensional protein structures of wild-type and mutant RB proteins (http://zhanglab.ccmb.med.umich.edu/I-TASSER).

### 2.4 *RB1* cDNA mutation analysis

TRIzol (Invitrogen, Carlsbad, CA, USA) was used to extract total RNA from the peripheral blood of healthy family members. Subsequently, the HiScript II 1st Strand cDNA Synthesis Kit (Vazyme, Jiangsu, China) was employed for synthesizing the first strand cDNA. cDNA fragments, spanning from exon 7 to exon 11, were amplified by using primers near the splice mutation site. The primer sequences were as follows: exon7-F: 5′-TCTCACCTCCCATGTTGCTC and exon11-R: 5′-AAGTCCATTAGATGTTACAAGTCCA. Amplified products were confirmed by Sanger sequencing. *GAPDH* was used as the reference gene.

### 2.5 RNA analysis

Total RNA extracted from the peripheral blood of family members was reverse transcribed into cDNA using the HiScript II Q RT SuperMix for qPCR (+gDNA wiper) sourced from Vazyme, Jiangsu, China. To determine the relative mRNA expression levels of the *RB1* genes, quantitative real-time (qRT) PCR was conducted utilizing the ChamQ SYBR qPCR Master Mix, also from Vazyme. GAPDH served as the reference gene to normalize the expression levels of the target gene. The gene expression levels were calculated using the 2^−ΔΔCT^ method. To ensure the reliability of our results, the qRT-PCR assays were repeated three times. To analyze the mRNA expression of the normal *RB1* gene, the upstream primer was set at exon 8, and the downstream primer was set at exon 9. The primer sequences of were as follows: *RB1* exon8-F: 5′-AACAGGAGTGCACGGATAGC and *RB1* exon9-R: 5′-AAGTCCATTAGATGTTACAAGTCCA; *GAPDH* F: 5′-GAAAGCCTGCCGGTGACTAA and *GAPDH* R: 5′-AGGAAAAGCATCACCCGGAG.

### 2.6 Immunohistochemical analysis

The tumor tissue was subjected to fixation using 4% paraformaldehyde, subsequently undergoing dehydration and embedding procedures. Afterward, 3-μM-thick tissue sections were meticulously prepared. After the paraffin sections were routinely dewaxed, the tissue sections were immersed in ethylenediaminetetraacetic acid (EDTA) antigen repair buffer (pH 9.0) for the purpose of antigen repair. These sections were then incubated in a 3% H_2_O_2_ solution and kept in a dark environment for 25 minutes. Following this, a drop of 3% bovine serum albumin (BSA) was added to the sections, which were then sealed at room temperature for 30 minutes and incubated at 4°C overnight with RB1 antibody (ProteinTech, Chicago, IL, USA; 10048-2-Ig). A goat anti-rabbit secondary antibody labeled with horseradish peroxidase (HRP) was added. The sections were colored with 3,3-diaminobenzidine (DAB), restained with hematoxylin, and sealed with neutral resin after dehydration. They were examined under a microscope.

### 2.7 Statistical analyses

Statistical analyses were performed using the GraphPad Prism software (GraphPad Software Inc., San Diego, CA, USA). Statistical significance between the two groups was determined using the independent samples *t*-test. Data are expressed as the mean ± standard deviation (SD). A p-value <0.05 was considered statistically significant, and the following symbols were used for p-values: *p < 0.05, **p < 0.01, ***p < 0.001, and ****p < 0.0001.

## 3 Results

### 3.1 Clinical reports

**Figure 1:**
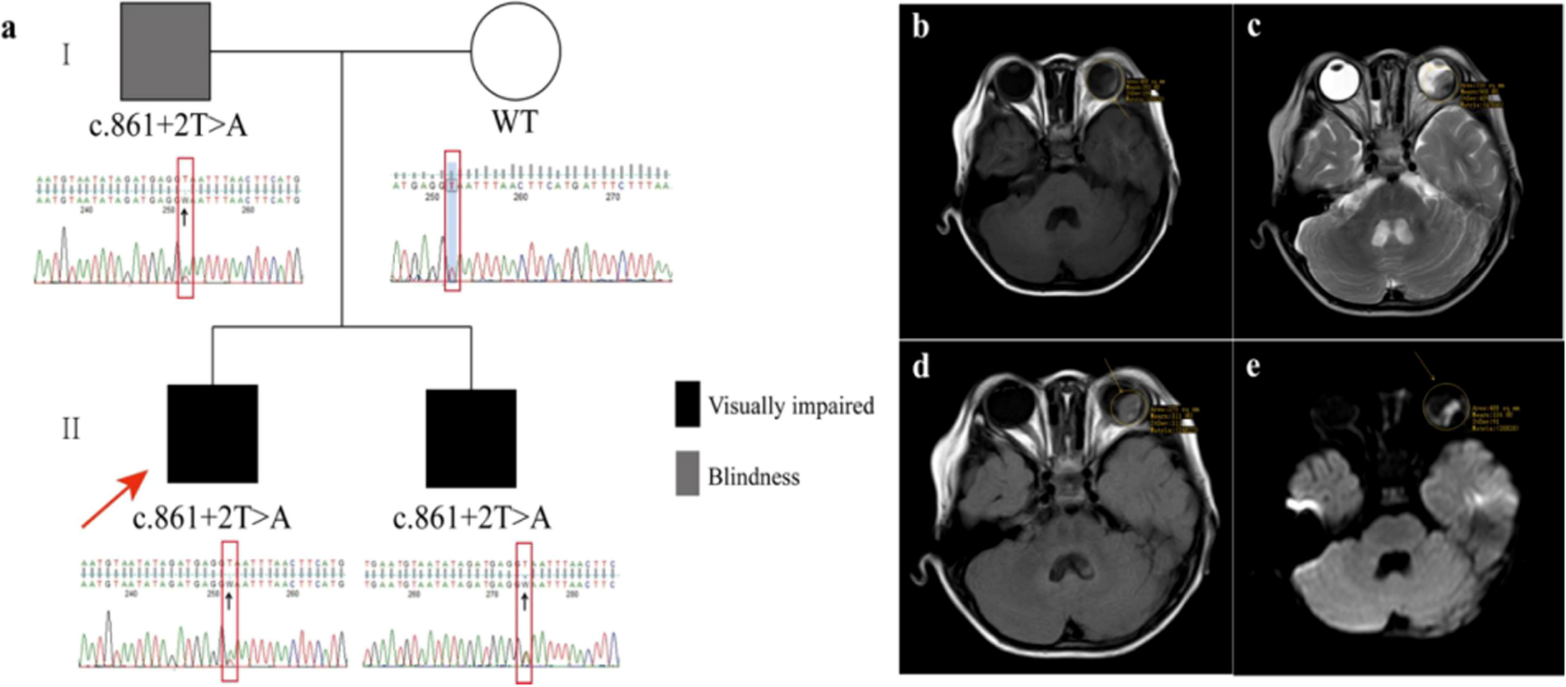
Clinical phenotype in the Chinese Tibetan pedigree with familial retinoblastoma. **(a)** Family pedigree and Sanger sequencing results. A germline donor splicing site mutation (c.861 + 2T>A) in *RB1* gene was confirmed by Sanger sequencing performed on peripheral blood in the proband. The father and younger brother of the proband both carried this mutation. *RB1* gene of the mother of the proband was wild type. **(b–e)** Images of cranial enhanced MRI. The results showed a mass shadow in the left eyeball, with mainly isointensity on T1-weighted images, uneven hypointensity on T2-weighted images, slight hyperintensity on Fluid Attenuated Inversion Recovery (T2-FLAIR), and hyperintensity on Diffusion-Weighted Imaging (DWI).

The proband was aged 2 years 11 months at the time of diagnosis and was referred from Tibet to the Ophthalmology Department of West China Second University Hospital due to leukocoria (**Figure 1a**). Fundus examination showed fundus mass and retinal protrusion with tumor implantation. Color Doppler ultrasound indicated a solid mass with calcification in the left fundus. Cranial enhanced MRI also showed a mass shadow in the left eyeball, with a size of approximately 1.8 cm × 1.2 cm × 1.7 cm, no abnormality of the left optic nerve signal, and no space occupying in the sellar region and pineal gland region (**Figures 1b–e**). After thorough deliberations by experts in ophthalmology, oncology, and radiology, the diagnosis of RB with intraocular phase D disease was confirmed, aligning with the International Intraocular Retinoblastoma Classification (IIRC) criteria (16). At the request of the parents, to preserve the eye, the patient was admitted to our department for chemotherapy and scheduled for regular follow-up fundus examinations in the ophthalmology department. The proband received six cycles of VEC (vincristine, etoposide, and carboplatin) chemotherapy. Fundus examinations and cranial enhanced MRI revealed a significant reduction of tumor mass in the left eye, prompting the discontinuation of medication based on the ophthalmologist’s evaluation for observation purposes. Subsequently, the patient could not attend regular fundus examinations after returning to their hometown. Five months post-treatment, the patient returned to West China Second University Hospital with symptoms of redness and swelling in the left eye. An orbital CT scan confirmed a tumor relapse. Due to the absence of normal lens and vitreous body structures in the left eye, and the indistinct boundary between the tumor tissue and extraocular muscles, an enucleation surgery was necessitated. The pathological findings were supportive of the diagnosis of RB. The proband underwent another four cycles of ICE (ifosfamide, carboplatin, and etoposide) chemotherapy and local radiation. However, the tumor relapsed at the primary site again and extensively involved the left parotid gland, bone marrow, spinal cord, and left forearm. His parents gave up treatment, and the proband died finally.

The proband’s father has been blind since childhood for unknown reasons (**Figure 1a**). Ophthalmic examination revealed no eyeballs in the orbit. Further inspections were not conducted for personal reasons. He was advised to undergo regular follow-up observations. Furthermore, the proband’s 5-month-old sibling underwent an ophthalmic examination and was diagnosed with bilateral retinoblastoma, presenting with leukocoria, approximately 2 months following the initial diagnosis of the proband (**Figure 1a**). Cranial enhanced MRI showed a mass shadow in both two eyeballs, with obvious involvement in the right eye accompanied by invasion of the optic papilla and disc regions. Due to his young age, a fundus examination was performed under general anesthesia. It was found that the vitreous cavity of the right eye was filled with tumors, and there was a white protrusion of approximately six optic discs in size in the retina below the left fundus. Fundus fluorescein angiography (FFA) in the left eye showed early filling of the tumor, dilation of capillaries, and strong fluorescence in the late stage; no leakage of fluorescein was observed. He was clinically diagnosed with RB, presenting with extraocular disease in the right eye and intraocular phase D disease in the left eye. Due to the same request for eye conservation, he was also admitted to our department for chemotherapy. After receiving six cycles of VEC chemotherapy, fundus examination revealed that a large area of tumor with partial calcification and new neoplasms above the optic disc was in the right eye fundus and new neoplasms above the temporal area and tumor atrophy in the lower retina in the left eye fundus. Another six cycles of VEC chemotherapy were given, and PET/CT was performed for evaluation with calcification on both sides of the right intraocular disc, with no signs of tumor residue or recurrence in both eyes and no signs of tumor metastasis in other parts of the body. Then, he was advised by his ophthalmologist to discontinue treatment and undergo regular follow-up. At the time of writing, 7 months after treatment completion, the patient is alive and disease-free.

### 3.2 Mutation analysis

**Figure 2:**
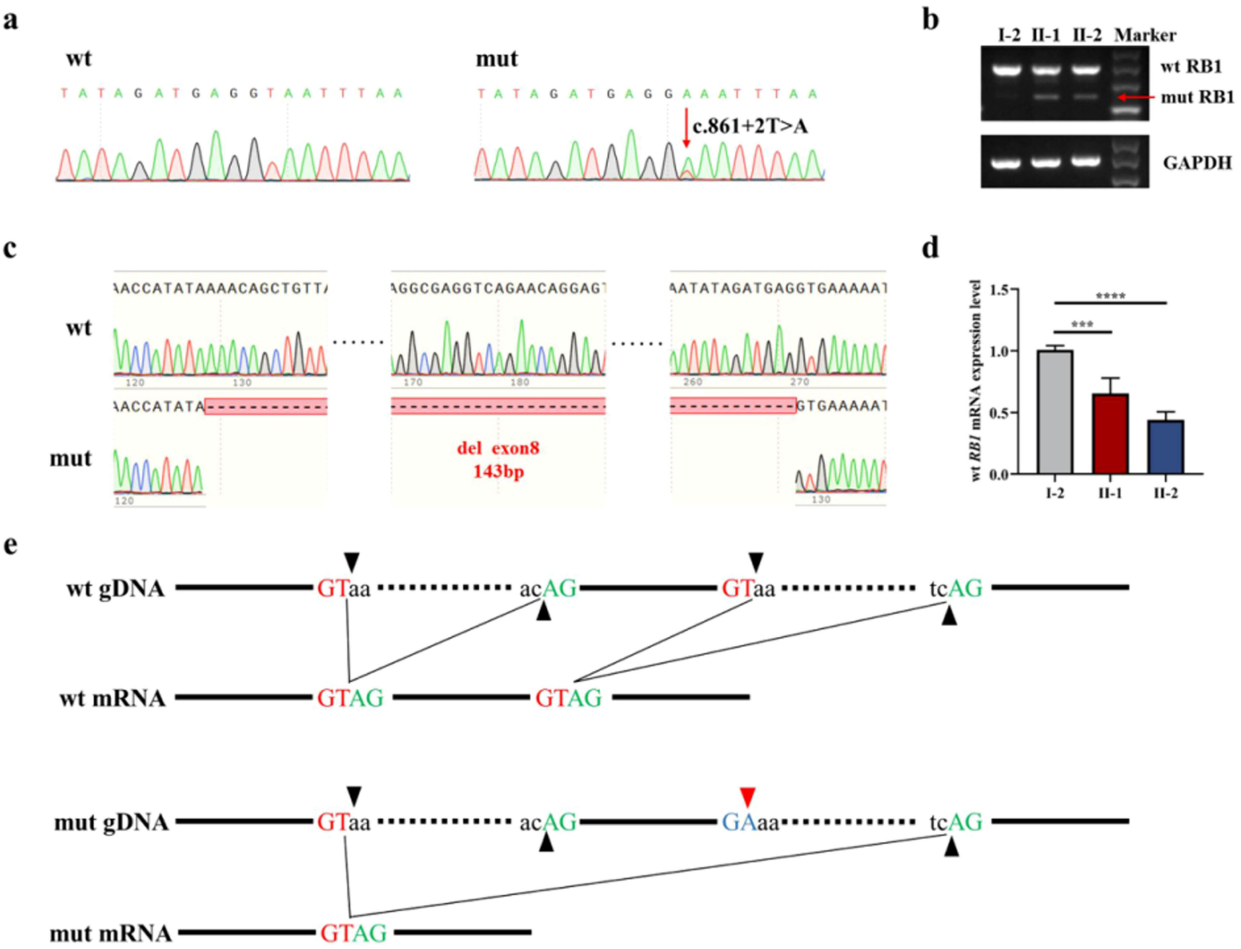
RB1 gene mutation analysis. **(a)** Sanger sequencing of genomic DNA amplification products. **(b)** Electrophoresis of cDNA amplification products, with red arrows indicating abnormal transcripts. **(c)** The Sanger sequencing of cDNA amplification products shows that the abnormal transcript caused by mutation is the deletion of exon 8. **(d)** Normal RB1 gene expression verification showed that the *RB1* gene in patients decreased. **(e)** Splicing diagram, with black arrows indicating splicing sites and red arrows indicating mutant bases. ***P<0.05 vs I-2. ****P<0.05 vs I-2.

A novel germline donor splicing site mutation (c.861 + 2T>A) of the *RB1* gene, located in NO.2 base behind exon 8, was identified by Sanger sequencing on the proband’s peripheral blood before chemotherapy (**Figure 2a**). The same donor splicing site mutation was also carried by the proband’s father and younger brother. This mutation has not been reported in the Genome Aggregation Database, the Exome Aggregation Consortium, and ClinVar Database at present. After *RB1* cDNA was amplified, the abnormal script was found to be smaller than the normal script (**Figure 2b**). The whole exon 8 with 143 bp was deleted in the abnormal script by Sanger sequencing (**Figure 2c**). Compared with that in normal individuals, the mRNA expression level of the normal *RB1* gene in patients decreased (**Figure 2d**). The splicing diagram is shown in **Figure 2e**.

### 3.3 Bioinformatics analysis

**Figure 3:**
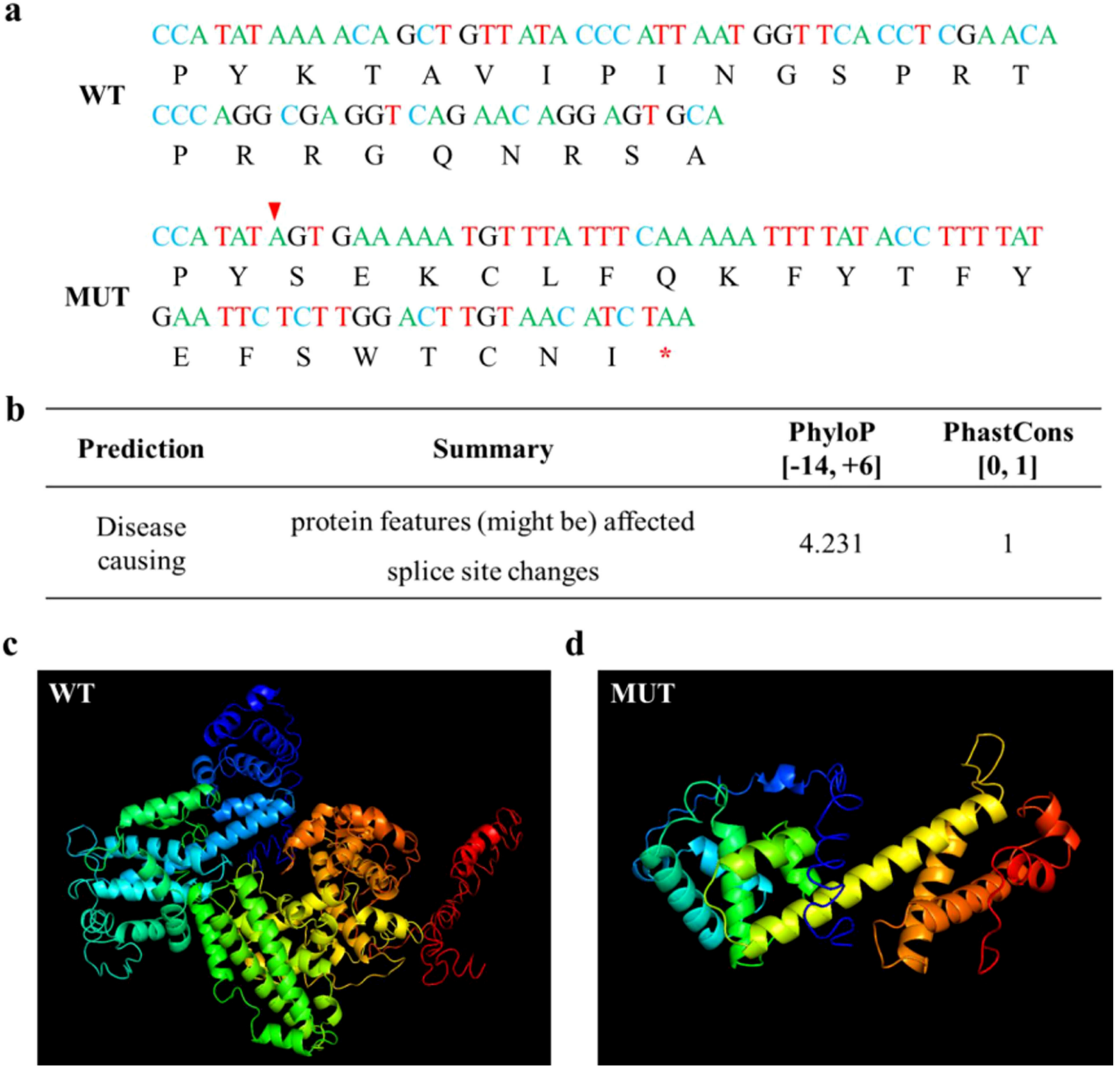
Bioinformatics analysis. **(a)** cDNA and coding protein sequences of wild and mutant RB1, with red arrows indicating abnormal splicing sites and red asterisks indicating termination codons. **(b)** Prediction results of MutationTaster. **(c)** Normal protein structure of RB1 predicted by I-TASSER. **(d)** Abnormal protein structure of RB1 predicted by I-TASSER.

Due to the c.861 + 2T>A splicing site mutation in the *RB1* gene, the entire exon 8 is missing, the codon is altered, and the termination codon occurs prematurely, resulting in the production of truncated proteins (**Figure 3a**). The prediction results of MutationTaster indicate that the base mutation has pathogenicity, and the protein features may be affected. Both the values of PhastCons and PhyloP indicate that the base site is highly conserved (**Figure 3b**). The three-dimensional structure predicted by I-TASSER indicates significant changes in the spatial structure of abnormal proteins after mutation (**Figures 3c, d**).

A somatic duplication mutation in exon 2 221–224 of the *RB1* gene, discovered by NGS on the proband’s tumor tissue after the enucleation surgery, induced a truncated protein at amino acid position 75. After searching the ClinVar Database (https://www.ncbi.nlm.nih.gov/clinvar) and Genome Aggregation Database (http://www.gnomad-sg.org/), it was found that the dupmutation mutation has also not been reported. According to the American College of Medical Genetics and Genomics (ACMG) 2015 guidelines, this mutation is classified as pathogenic, supported by PVS1+PS2. Moreover, the results of CMA showed that mosaic gains of two to three times were identified in 1q21.1-q44 and 2p25.3-p24.1, with the latter variation involved in the *MYCN* gene.

### 3.4 Immunohistochemical analysis

**Figure 4:**
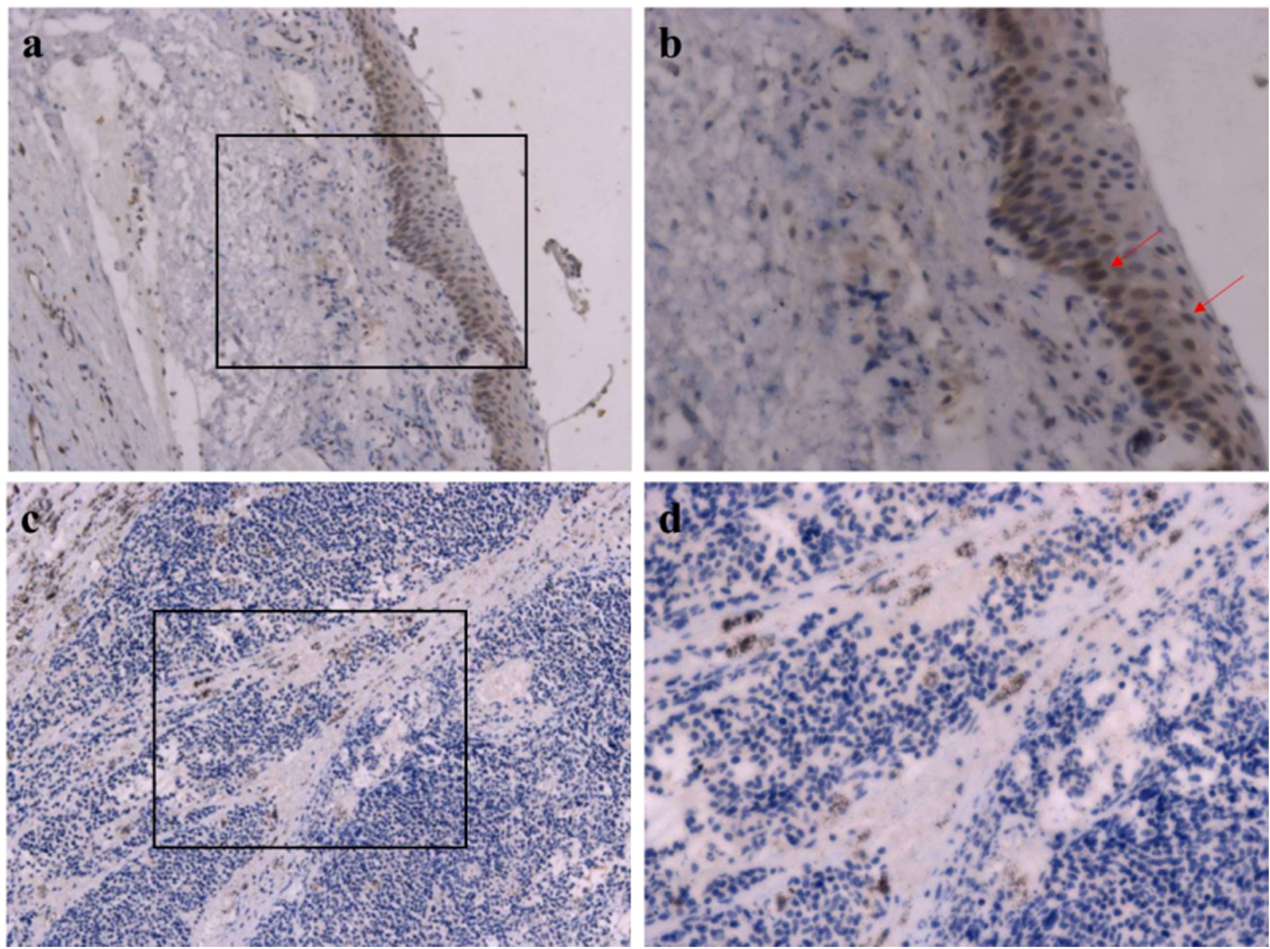
Immunohistochemical images of RB1. **(a, b)** Normal tissue, intense and diffuse expression in normal ocular tissue; red arrow indicates RB1-positive cells. **(c, d)** Tumor tissue, absence of expression in the tumor cells. Original magnification: **(a, c)** ×200; **(B, D)** ×400.

Immunohistochemistry evaluation showed that RB1 was intensely and diffusely expressed in normal ocular tissue (**Figures 4a, b**) while lacking expression in the tumor cells (**Figures 4c, d**).

## 4 Discussion

Retinoblastoma management requires individualized treatment based on International Classification of Retinoblastoma (ICRB) staging, germline mutation status, family psychosocial factors and cultural beliefs, and available institutional resources (4). Being a rare malignancy, data on retinoblastoma outcomes are sparse, especially from low-income and middle-income countries. According to the data from the Global Retinoblastoma Study Group, mean overall and disease-free survival rates vary considerably depending on socioeconomic status, ranging from less than 50% to over 90%. In China, approximately 910–1,100 new retinoblastoma cases are diagnosed annually (17, 18). Because of rising awareness of this disease in recent years, the disease-specific survival rates were 81%, 83%, and 91% in 1989–2008, 2009–2013, and 2014–2017, respectively, showing an increasing trend (19).

Genetic implication for more than 97% of retinoblastoma is the biallelic inactivation of the *RB1* gene, preventing the production of functional RB proteins (20). Hereditary RB accounts for 40%–50% of all cases, with only 5%–10% of patients having a positive family history (14). Therefore, genetic testing and counseling are the internationally accepted convention for RB and integral to the precise and comprehensive management of RB (14, 20, 21). However, due to its unavailability or unreliability locally, or cost barriers, genetic testing is often not a standard part of retinoblastoma management in most centers around the world, particularly in developing countries. In this study, the proband was initially clinically diagnosed to be unilateral RB but was unable to undergo genetic screening due to unaffordable testing costs. After the recurrence of the proband’s disease, the *RB1* gene mutation screening of peripheral blood, and NGS and CMA of tumor tissue for the proband were conducted free of charge by our center. A novel germline donor splicing site mutation (c.861 + 2T>A) of the *RB1* gene was identified by Sanger sequencing on the proband’s peripheral blood, a somatic duplication mutation (c.221_224dup, p.W75Cfs*36) in exon 2 was discovered by NGS, and mosaic gains of two to three times were identified in 1q21.1-q44 and 2p25.3-p24.1 by CMA on the proband’s tumor tissue. The splice-site alteration of c.861 + 2T>G has been reported in families with retinoblastoma (22, 23). Given the involvement of splice-site alterations in the aforementioned cases, it is reasonable to postulate that the resultant protein structural modifications would exhibit comparable structural perturbations to those documented in the published case reports. Then, we further investigated the medical history and locus-specific amplification of the germline mutation of the family members. The proband’s father was blind for unknown reasons, and the proband’s younger brother was screened and diagnosed with bilateral RB. Both of them carried the same germline *RB1* gene mutation. Therefore, both the proband and his younger brother were highly suspected to be familial RB. A secondary “hit” is involved in the genetic underpinnings of heritable RB primarily, and it results in a second mutation in the *RB1* gene or epigenetic alterations leading to gene silencing. Mutations that cause exon deletion or addition such as splice error and large rearrangement mutations are often observed in these patients as in the present cases (24). Interestingly, hereditary retinoblastoma demonstrates incomplete penetrance because of *RB1* gene modifiers or only partial inactivation. Therefore, some cases of hereditary retinoblastoma exhibit a much lower penetrance rate. Moreover, mosaicism for *RB1* mutations can also contribute to unilateral or bilateral manifestation in RB cases. It is reported that bilateral disease occurs in 40% of RB patients, whereas unilateral manifestation is detected in 60% of cases (24). Hence, in this case, the proband has unilateral RB, but his sibling has bilateral RB. In addition, the identification of mutations in FGFR4, NQO1, ACADS, CX3CR1, GBE1, KRT85, and TYR are reported potential alternative pathways or mechanisms that may contribute to the pathogenesis of the disease in patients who lack germline *RB1* gene mutations (24). However, there were no other splice mutations included in this case. Understanding these nuances in patients without *RB1* gene mutations could offer new insights into the biology of RB and potentially lead to novel therapeutic approaches.

Akdeniz Odemis et al. found that the family history may suggest that the cases are hereditary in most cases (24). In our case, due to economic poverty and social factors, the father did not undergo an examination to determine the cause of his blindness. It was indicated that the accuracy of the medical history is crucial for avoiding stigmas such as cancer being considered a “death toll”. This case also emphasizes the importance of genetic counseling and educating the population that cancer is not fatal, early diagnosis is possible, or cancer can be totally prevented in some cases.

*RB1* gene harbors a large spectrum of pathogenic variants, with approximately 2,500 discovered so far, with more than 500 different somatic or germline mutations resulting in *RB1* inactivation (14). Common genetic variations include chromosomal rearrangements, large exonic deletions, hypermethylation of the gene promoter region, small-length mutations, and single-nucleotide substitutions (14, 21). The majority of germline mutations that have been identified in familial RB are nonsense or frameshift mutations within exons 2–25 (21, 25). Out-of-frame exon skipping due to splice-site variants is also commonly found, resulting in truncated proteins as well (26–28). Approximately 40 pathogenic splice donor site mutations have been reported in retinoblastoma by the NCBI ClinVar database (https://www.ncbi.nlm.nih.gov/clinvar) and Genome Aggregation Database (http://www.gnomad-sg.org/). The donor splicing site mutation in our study has not been reported at present. According to the ACMG 2015 guidelines, the pathogenicity of this mutation was unclear (PVS1, PM2, and PM6), and further validation tests were carried out. After *RB1* cDNA was amplified, the abnormal script was found to be smaller than the normal script. The whole exon 8 with 143 bp was deleted in the abnormal script by Sanger sequencing. The mRNA expression level of the normal *RB1* gene in patients decreased, and the termination codon occurred prematurely, resulting in the production of truncated proteins. Meanwhile, a novel somatic duplication mutation (c.221_224dup, p.W75Cfs*36) is in another *RB1* allele of the proband. Immunohistochemistry staining on the tissue of enucleation showed the absence of RB1 expression in the tumor cells. Based on the above results and his family history, the diagnosis of familial RB was confirmed.

The incidence of orbital recurrence and metastatic disease is <1% in advanced countries while approximately 9%–11% according to limited reports from developing countries (29–31). The risk of local recurrence increases in the setting of high-risk histologic features, such as tumor invasion into the sclera, optic nerve, anterior chamber, and choroid, but distant metastasis is exceedingly rare and is usually confined to the central nervous system (32). Metastatic diseases are usually reported to occur within the first 1–2 years after initial diagnosis (31). The proband in our study experienced disease recurrence approximately 1 year after diagnosis and metastasis to multiple intracranial and extracranial sites after salvage chemotherapy and local radiation, ultimately giving up treatment and dying. Despite advanced retinoblastoma in both eyes, the proband’s younger brother successfully preserved his eyesight following treatment. To date, both the proband’s father and younger brother are under close follow-up care.

In conclusion, RB is a mostly curable cancer if diagnosed and treated early. The primary goals of RB treatment are to protect life, prevent metastatic disease, and then preserve the globe and useful vision. With modern treatment protocols and early disease identification, success rates of disease-free globe and eye preservation can reach up to 100%. However, the treatment of advanced cases remains complex, requiring aggressive chemotherapy and/or external radiation. Advances in the deep understanding of the molecular drivers in the RB pathway will provide opportunities to explore novel target drugs, improve bioavailability, and reduce chemotoxicity.

## Data Availability

The datasets presented in this study can be found in online repositories. The names of the repository/repositories and accession number(s) can be found in the article/**Supplementary Material**.
