# Supplementary material for: Case Report: A novel germline donor splicing site mutation of RB1 gene in a Chinese Tibetan pedigree with familial retinoblastoma
Source: Front Oncol. 2025 May 20;15:1525035. doi: 10.3389/fonc.2025.1525035 (PMC12130007; doi:10.3389/fonc.2025.1525035)
Supplement: Supplementary file 1 [file Table1.doc]

**Table 1. The primer sequence of Exon1 to Exon27 of RB1**

|  | **Primer name** | | **Primer sequence** | **Size（bp）** |
| --- | --- | --- | --- | --- |
| Exon1 | Forward | RB1-exon1-F | GACTTAGCGTCCCAGCCC | 636 |
|  | Reverse | RB1-exon1-R | GTTGAAGCCGAGACCCCT |  |
| Exon2 | Forward | RB1-exon2-F | ACAGAAGTGTTTTGCTGCTTTGA | 534 |
|  | Reverse | RB1-exon2-R | CTCTGGGTAATGGAATTATTATTAGC |  |
| Exon3 | Forward | RB1-exon3-F | CAGTTTTAACATAGTATCCAG | 281 |
|  | Reverse | RB1-exon3-R | AGCATTTCTCACTAATTCAC |  |
| Exon4 | Forward | RB1-exon4-F | GTAGTGATTTGATGTAGAGC | 305 |
|  | Reverse | RB1-exon4-R | CCCAGAATCTAATTGTGAAC |  |
| Exon5 | Forward | RB1-exon5-F | AGGTGGATCAGCTGGGTGTT | 612 |
|  | Reverse | RB1-exon5-R | TGCCTGTAACAGACCCTCAA |  |
| Exon6 | Forward | RB1-exon6-F | GAAACACCCAAAAGATATATCTGG | 326 |
|  | Reverse | RB1-exon6-R | CCAAGGTTGTTTCTAGTACCAG |  |
| Exon7 | Forward | RB1-exon7-F | CCTGCGATTTTCTCTCATAC | 256 |
|  | Reverse | RB1-exon7-R | ATGTTTGGTACCCACTAGAC |  |
| Exon8 | Forward | RB1-exon8-F | AGTAGTAGAATGTTACCAAG | 380 |
|  | Reverse | RB1-exon8-R | TACTGCAAAAGAGTTAGCAC |  |
| Exon9 | Forward | RB1-exon9-F | TGCATTGTTCAAGAGTCAAG | 222 |
|  | Reverse | RB1-exon9-R | AGTTAGACAATTATCCTCCC |  |
| Exon10 | Forward | RB1-exon10-F | TCTTTAATGAAATCTGTGCC | 291 |
|  | Reverse | RB1-exon10-R | GATATCTAAAGGTCACTAAG |  |
| Exon11 | Forward | RB1-exon11-F | GAGACAACAGAAGCATTATAC | 245 |
|  | Reverse | RB1-exon11-R | CGTGAACAAATCTGAAACAC |  |
| Exon12 | Forward | RB1-exon12-F | TAGAGACAAGTGGGAGGCAGT | 631 |
|  | Reverse | RB1-exon12-R | ACCTGGAATTGAAGATCGAGCAA |  |
| Exon13 | Forward | RB1-exon13-F | CTTATGTTCAGTAGTTGTGG | 342 |
|  | Reverse | RB1-exon13-R | TATACGAACTGGAAAGATGC |  |
| Exon14 | Forward | RB1-exon14-F | CATAATTGTGATTTTCTAAAATAGC | 237 |
|  | Reverse | RB1-exon14-R | TGGCCAGGATGATCTTGATGC |  |
| Exon15-16 | Forward | RB1-exon15-F | CAATGCTGACACAAATAAGG | 366 |
|  | Reverse | RB1-exon16-R | AGCATTCCTTCTCCTTAACC |  |
| Exon17 | Forward | RB1-exon17-F | AAAAATACCTAGCTCAAGGG | 339 |
|  | Reverse | RB1-exon17-R | TGTTAAGAAACACCTCTCAC |  |
| Exon18 | Forward | RB1-exon18-F | TGTACCTGGGAAAATTATGC | 340 |
|  | Reverse | RB1-exon18-R | CTTTATTTGGGTCATGTACC |  |
| Exon19 | Forward | RB1-exon19-F | ATAATCTGTGATTCTTAGCC | 273 |
|  | Reverse | RB1-exon19-R | AAGAAACATGATTTGAACCC |  |
| Exon20 | Forward | RB1-exon20-F | AAAGAGTGGTAGAAAAGAGG | 335 |
|  | Reverse | RB1-exon20-R | CAGTTAACAAGTAAGTAGGG |  |
| Exon21 | Forward | RB1-exon21-F | GCCTTGGTGATTTGCATTTTG | 401 |
|  | Reverse | RB1-exon21-R | CTCATTAATGAATAAATGAGATC |  |
| Exon22 | Forward | RB1-exon22-F | TTACTGTTCTTCCTCAGACATTC | 263 |
|  | Reverse | RB1-exon22-R | GCTTCGAGGAATGTGAGGTATTGG |  |
| Exon23 | Forward | RB1-exon23-F | ATCTAATGTAATGGGTCCAC | 287 |
|  | Reverse | RB1-exon23-R | CTTGGATCAAAATAATCCCC |  |
| Exon24 | Forward | RB1-exon24-F | GAATATAGTTTGTCAGTGGTTC | 277 |
|  | Reverse | RB1-exon24-R | GTGTTTGAATAACTGCATTTGG |  |
| Exon25 | Forward | RB1-exon25-F | GGTTGCTAACTATGAAACAC | 297 |
|  | Reverse | RB1-exon25-R | AGAAATTGGTATAAGCCAGG |  |
| Exon26 | Forward | RB1-exon26-F | AGTAAGTCATCGAAAGCATC | 209 |
|  | Reverse | RB1-exon26-R | AACGAAAAGACTTCTTGCAG |  |
| Exon27 | Forward | RB1-exon27-F | CGCCATCAGTTTGACATGAG | 237 |
|  | Reverse | RB1-exon27-R | CAGTCACATCTGTGAGAGAC |  |
